# Can Youthful Mesenchymal Stem Cells from Wharton’s Jelly Bring a Breath of Fresh Air for COPD?

**DOI:** 10.3390/ijms18112449

**Published:** 2017-11-18

**Authors:** Andrzej M. Janczewski, Joanna Wojtkiewicz, Ewa Malinowska, Anna Doboszyńska

**Affiliations:** 1 Department of Pulmonology, Faculty of Heath Sciences, University of Warmia and Mazury in Olsztyn, Jagiellońska 78, 10-357 Olsztyn, Poland; ewamalinowska@windowslive.com (E.M.); anna.doboszynska@wp.pl (A.D.); 2 Department of Pathophysiology, Faculty of Medicine, University of Warmia and Mazury in Olsztyn, Warszawska 30, 10-082 Olsztyn, Poland; joanna.wojtkiewicz@uwm.edu.pl; 3 Laboratory for Regenerative Medicine, Faculty of Medicine, University of Warmia and Mazury in Olsztyn, Warszawska 30, 10-082 Olsztyn, Poland; 4 Foundation for the Nerve Cells Regeneration, Warszawska 30, 10-082 Olsztyn, Poland

**Keywords:** chronic obstructive pulmonary disease, Wharton’s jelly, mesenchymal stem cells, anti-inflammatory effects, immunomodulation, therapeutic applications

## Abstract

Chronic obstructive pulmonary disease (COPD) is a major global cause of morbidity and mortality, projected to become the 3rd cause of disease mortality worldwide by 2020. COPD is characterized by persistent and not fully reversible airflow limitation that is usually progressive and is associated with an abnormal chronic inflammatory response of the lung to noxious agents including cigarette smoke. Currently available therapeutic strategies aim to ease COPD symptoms but cannot prevent its progress or regenerate physiological lung structure or function. The urgently needed new approaches for the treatment of COPD include stem cell therapies among which transplantation of mesenchymal stem cells derived from Wharton’s jelly (WJ-MSCs) emerges as a promising therapeutic strategy because of the unique properties of these cells. The present review discusses the main biological properties of WJ-MSCs pertinent to their potential application for the treatment of COPD in the context of COPD pathomechanisms with emphasis on chronic immune inflammatory processes that play key roles in the development and progression of COPD.

## 1. Introduction

Chronic obstructive pulmonary disease (COPD) is a common, preventable and treatable disease characterized by persistent respiratory symptoms and airflow limitation consequent to airway and/or alveolar abnormalities usually caused by significant exposure to noxious particles or gases [1]. COPD is currently one of the leading global causes of morbidity and mortality, projected to become the 3rd cause of disease mortality worldwide by 2020, bringing a substantial and increasing medical, economic, and social burden [1,2]. Chronic airflow limitation in COPD is caused by a combination of small airways disease (obstructive bronchiolitis) and parenchymal destruction (emphysema), the relative contributions of which vary from person to person with respect to concurrent occurrence and/or rates of evolvement over time. Structural and functional changes caused by chronic inflammation include narrowing or loss of small airways, mucus hypersecretion, mucociliary dysfunction, and destruction of the lung parenchyma that leads to the loss of alveolar attachments to the small airways and decreases lung elastic recoil. In turn, these changes diminish the ability of the airways to remain open during expiration [1,3,4,5]. Specific pathomechanisms of COPD remain incompletely understood but are thought to involve complex interactions of noxious airborne agents, such as cigarette smoke, with genetic predispositions involving multiple genes [6,7], which lead to structural and functional changes in the airways mediated primarily by a pathogenic triad: inflammation, oxidative stress, and protease-antiprotease imbalance [8]. The multifaceted and often mutually amplifying interactions among numerous components of this pathogenic triad, i.e., inflammation, innate and acquired immunity, and tissue destruction and repair [3,4,5,8], underlie the usually escalating character of COPD and slow progress in comprehensive understanding of the disease.

Inflammatory and immune processes appear to play key roles in the development and progression of COPD and are self-perpetuating, i.e., persist despite the cessation of smoking [9,10] in large part by the actions of immune inflammatory pathways [3,4,5,11,12]. Breaking the vicious cycle maintained by these pathways in COPD requires novel therapeutic strategies as currently available therapies enable easing COPD symptoms but cannot prevent its progress or regenerate physiological lung structure or function. The urgently needed new approaches for the treatment of COPD include stem cell therapies among which transplantation of mesenchymal stem cells (MSCs) derived from Wharton’s jelly (WJ-MSCs) emerges as a promising and yet unexplored therapeutic strategy for COPD. This review discusses the main biological properties and mechanisms of action of MSCs in general and WJ-MSCs in particular in the context of their potential application for the treatment of COPD with emphasis on their effects on chronic inflammatory and immune processes.

## 2. Inflammation and Airway Remodeling in COPD

Exposure to noxious particles or gases such as cigarette smoke causes airway inflammatory response which, depending on the extent of exposure and genetic predisposition, may cascade into a multifaceted, self-propagating and evolving inflammatory phenotype characteristic of COPD [3,4,5,11,12]. The inflammatory process in the lungs of COPD patients involves a specific pattern that comprises accumulation (particularly in small airways) of macrophages, neutrophils, CD8+ cytotoxic lymphocytes, and other cells which release multiple pro-inflammatory mediators, growth factors, and mitogenic and pro-fibrotic factors. These immune inflammatory cells cause the destruction and remodeling of the pulmonary tissue both directly and through interactions with structural cells of the airways (epithelial cells and fibroblasts), lung parenchyma (alveolar epithelial cells), and pulmonary vasculature (endothelial cells), by stimulating lung resident cells to secrete other mediators sustaining the inflammatory process [13,14,15,16]. 

Among inflammatory immune cells primarily implicated in the pathomechanisms of COPD (i.e., macrophages, neutrophils, monocytes, dendritic cells, natural killer cells, mast cells, T cells, and B cells), macrophages appear to play one of key roles as they are directly activated by cigarette smoke extract and secrete inflammatory proteins that coordinate the inflammatory process in COPD via effects on a number of immune cells including neutrophils, monocytes, and CD8+ lymphocytes [3,4,5]. Macrophages also release several humoral factors (or modify their release from other cells) involved in COPD pathomechanisms, including cytokines, other chemokines, and elastolytic enzymes [5]. However, macrophages may also inhibit inflammatory processes and facilitate tissue repair depending on their polarization, e.g., via MSC–macrophage crosstalk modifying macrophage polarization towards anti-inflammatory M_2_ rather than pro-inflammatory M_1_ class [17].

Other immune cells that are thought to play main roles in the induction and perpetuation of chronic inflammation, and the consequent lung destruction in COPD, are neutrophils, T lymphocytes, and B lymphocytes. Smoking-induced accumulation and activation of circulating and lung-resident neutrophils, as well as the resulting destruction of the elastic matrix of the alveoli by proteases and oxygen-derived free radicals released by these cells, is a prominent feature of COPD [3,5,18]. While participation of T cells in COPD pathomechanisms is well established, precise roles of specific T-cell fractions, including memory CD8+ T cells, T regulatory cells (Tregs), T helper cells (Th1, Th2, Th17), have yet to be fully elucidated. The same applies to the role of B cells. Available data, recently reviewed by Bagdonas and colleagues [5], shows (1) that Th1-type cytokines (primarily interferon-γ; INF-γ) participate in perpetuating autoimmune responses and in the resulting excessive inflammatory response associated with tissue damage in COPD, (2) that Th (CD4+) cells upon activation release cytokines and orchestrate the activity of other inflammatory and related cells, and (3) that T cells from cigarette smoke-exposed mice are able to transfer the disease process (i.e., tissue destruction) to unexposed mice, demonstrating a cigarette smoke-induced, T cell-driven autoimmune mechanism in COPD development. Furthermore, the T cell-mediated inflammatory response is already present in mild COPD, but considerably increases with disease severity, suggesting that the initial immune response becomes self-perpetuating, in part at least, because of endogenous autoantigens generated by inflammatory and oxidative lung injury [19]. Recent studies, reviewed by Rovina et al. [12], have also shown that cigarette-smoke-driven agents (including antigens, lung tissue breakdown products, and/or autoantigens) may elicit adaptive immune responses in the lungs of COPD patients with the participation of cytotoxic CD8+ T cells, Th1, and Th17 CD4+ cells (and B cell responses with antibody production), and that the extent of airflow limitation and emphysema in COPD correlates with the number of pulmonary CD8+ T cells, which, upon activation, release proteolytic enzymes causing the death of structural cells by apoptosis or necrosis. Interestingly, accumulating evidence suggests that emphysema in COPD may be consequent not only to immune inflammatory processes in the lung parenchyma but also to inflammation- and fibrotic-remodeling-dependent obstruction of small airways [15,20,21]. Taken together, the aforementioned data indicate that anti-inflammatory and immunomodulatory effects of MSCs (discussed in the next section) have a potential to impede both major phenotypic subsets of COPD, chronic bronchitis, and emphysematous lung destruction.

## 3. Mesenchymal Stem Cells

Mesenchymal stem cells, also defined as multipotent stromal cells, are a heterogeneous population of cells that can be harvested from many tissues including bone marrow, adipose tissue, and the umbilical cord. MSCs have the capacity to differentiate into a variety of mesodermal cell lineages and can be expanded and differentiated ex vivo [22]. MSCs have been defined by the Mesenchymal and Tissue Stem Cell Committee of the International Society for Cellular Therapy by three criteria: (1) adherence to plastic under standard culture conditions; (2) an expression of surface markers CD105, CD73, and CD90, and lack of surface expression of hematopoietic markers CD45, CD34, CD14, CD11b, CD79, CD19, and class II human leukocyte antigen-DR (HLA-DR); and (3) an ability to differentiate into adipocytes, chondrocytes, and osteocytes in vitro [23]. Besides these unified and minimal criteria, physically different populations of MSCs isolated from adult tissues (such as bone marrow or adipose tissue) and fetal tissues (such as the umbilical cord) exhibit distinctively different proteomes, immunophenotypes, and profiles of the secreted cytokines/chemokines, growth factors, and enzymes [24]. A well-documented ability of human MSCs to largely avoid allorecognition and to generate a local immunosuppressive and tissue-regenerating microenvironment (by secreting cell signaling molecules and/or by cell-to-cell interactions) has generated widespread interest in the fields of cell-based immunomodulation and regenerative medicine [25,26,27].

## 4. Anti-Inflammatory and Immunomodulatory Effects of MSCs in COPD

Numerous studies in experimental models have evidenced beneficial effects of anti-inflammatory, immunomodulatory, and tissue repair actions of MSCs on major pathways involved in the pathogenesis of COPD (for recent reviews, see [28,29,30,31,32]). These preclinical studies have laid the foundation of the recent early-phase (I/II) clinical trials examining the effects of MSC transplantation in patients with COPD [33,34,35]. 

Native MSCs home specifically to the sites of tissue injury and inflammation by the action of chemokines, cytokines, and growth factors released upon injury, which also provide migratory cues for systemically or locally administered exogenous MSCs [36]. Homing and migration of MSCs to a target tissue have been defined by Karp and Leng Teo [37] as the arrest of MSCs within the vasculature of the respective tissue followed by transmigration across the endothelium. A convenient feature in the treatment of lung diseases with systemically infused MSCs is that they are initially entrapped in the lung vasculature [38,39]. However, relatively little is known about the specific mechanisms directing stem cell transmigration, a critical step for anti-inflammatory and immuno-modulatory effects of MSCs within the injury and reparative environment. Studies examining engraftment and differentiation of exogenous MSCs into lung tissue cells showed limited viability of this approach in regenerative therapy of lung disease [40,41,42,43].

Research evidence accumulated over the past decade [27,30,32,44,45] indicates that immunomodulatory, anti-inflammatory, and tissue repair effects of MCSs, i.e., effects crucial for potential treatment of diseases such as COPD, are mediated in large part through paracrine factors that influence both the immune inflammatory cells and structural lung cells. These paracrine effects are mediated via secretion of anti-inflammatory, immunomodulatory, anti-apoptotic, and angiogeneic factors. Recent studies have demonstrated that MSC treatment attenuates inflammation by increasing levels of anti-inflammatory mediators such as prostaglandin E2 [46] and interleukin (IL)-10 [47], while decreasing levels of inflammatory mediators such as interleukin (IL)-1β, IL-6, tumor necrosis factor (TNF)-α, INF-γ, indoleamine-pyrrole 2,3-dioxygenase, hepatocyte growth factor (HGF), nitric oxide, and chemokine (C-C motif) ligand 2 [48,49]. The complexity of MSC signaling is exemplified by the effects on neutrophils, mediated primarily via IL-6. Specifically, MSCs have been shown to protect neutrophils from apoptosis while harnessing their inflammatory potential by inhibition of reactive oxygen species production without impairing phagocytosis and chemotaxis [50], and to limit neutrophil recruitment to inflamed endothelium [51].

**Figure 1:**
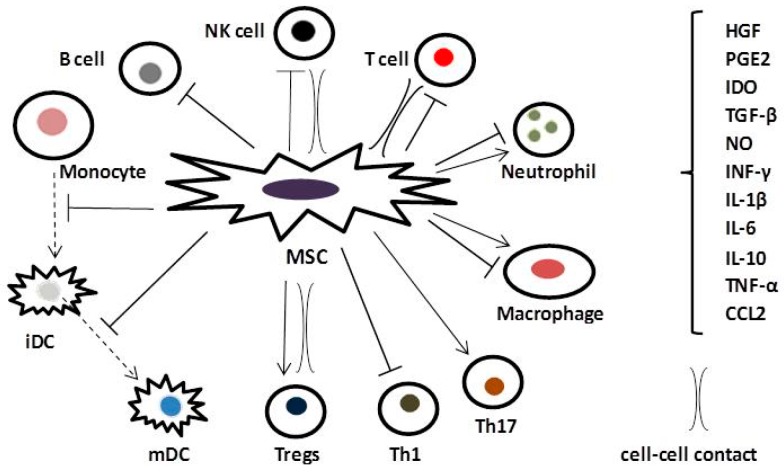
Effects of MSCs on generation, maturation, and proliferation of monocyte-derived immature (iDC) and mature dendritic cells (mDC) mediated via prostaglandin E2 (PGE2), transforming growth factor-β (TGF-β), interleukin (IL)-6; stimulation of T regulatory cells (Tregs) via PGE2, TGF-β, IL-10, cell-cell contact; inhibition of T helper (Th) cells subtype 1 (Th1) via PGE2; stimulation of Th17 cells via PGE2; macrophage differentiation via PGE2; multifaceted regulation of neutrophils via IL-6; downregulation of T cells induction, proliferation and function via PGE2, TGF-β, IL-1β, IL-6, IL-10, interferon-γ (INF-γ), indoleamine-pyrrole 2,3-dioxygenase (IDO), hepatocyte growth factor (HGF), nitric oxide (NO), cell–cell contact; inhibition of natural killer (NK) cells via PGE2, TGF-β, INF-γ, cell–cell contact; inhibition of B cells via INF-γ, chemokine (C-C motif) ligand 2 (CCL2); dash arrow: cell differentiation; solid arrow: stimulation; T-bar: inhibition.

Local signaling by MSCs is also mediated through cell–cell contact. Several recent studies [27,32,44,45] have indicated that cell contact plays a crucial part in the immunomodulatory effects of MSCs. The reported cell contact-dependent mechanisms of MSCs actions, mediated by secretion of cell adhesion molecules (i.e., CD274/programmed death ligand 1, vascular cell adhesion molecule-1 and galectin-1), reduce proliferation and survival of T cells, increase T_2_ fraction of Tregs [52,53], and suppress natural killer (NK) cells [54]. Cytokines, particularly INF-γ, have been shown to prime immunomodulatory capacity of MSCs including facilitation of cell-cell contacts and upregulation of adhesion molecules [55,56]. Novel findings also demonstrate cell-contact-dependent transfer of cellular materials (such as proteins, nucleic acids, and cell organelles including mitochondria) from MSCs to inflammatory immune cells and to structural cells of the lungs [27,32,45]. Collectively, current data demonstrate that both soluble factors and cell contact are indispensable in a multilayered and often reciprocal immunomodulation by MCSs. The anti-inflammatory and immunomodulatory effects of MSCs mediated through interactions with inflammatory cells implicated in the pathomechanisms of COPD are illustrated in Figure 1.

In addition to the anti-inflammatory, immunomodulatory, and tissue-repair actions of MSCs reviewed in the previous section, studies in animal models of COPD (Table 1) have revealed other mechanisms by which MSCs alleviate airway inflammation and emphysema, such as inhibition of fibrosis, apoptosis and mucus production, and enhanced angiogenesis.

## 5. Current Status of MSC Therapy for the Treatment of COPD

Based on robust, promising results of preclinical reports using MSCs in chronic inflammatory and immune-mediated conditions, including animal models corresponding to COPD [29,30,31,78], there are currently a number of Phases I–II clinical studies listed at ClinicalTrials.gov [79], examining the safety and efficacy of systemic administrations of autologous stem cells from bone marrow (BM-MSCs), adipose tissue (AT-MSCs), and allogeneic BM-MSCs in COPD patients. Thus far, two of these investigations have been completed. The first one, a multicenter, double-blind, placebo-controlled Phase II trial of systemic administration of allogeneic BM-MSC preparation (Prochymal; Osiris Therapeutics Inc., Columbia, MD, USA) in 62 patients with moderate-severe COPD, has demonstrated safety with no acute infusion toxicity and no attributable mortality or serious adverse events over a subsequent two-year follow-up period, and a significant early decrease in the systemic inflammatory marker C-reactive protein in a sub-population of MSC-treated patients with elevated C-reactive protein levels at study onset [80]. The other study completed thus far tested the effects of autologous systemic infusion of bone marrow mononuclear cells in four patients with advanced COPD (stage IV dyspnea), reporting safety and a lack of adverse effects, an improvement in functional tests (spirometry) indicative of slowing down in the process of pathological degeneration, and a significant improvement in patients’ quality of life [81]. Importantly, and consistently with the results of several Phases I–II clinical studies using systemic infusions of MSCs in patients with other diseases (see below), these clinical trials have demonstrated the safety of MSC use including multiple MSC infusions in patients with COPD [80,81]. However, clinically relevant therapeutic effects of these studies were rather limited compared to the promising results of preclinical investigations using MSCs in animal models of COPD (Table 1). Clearly, experimental models mimic only some aspects of human disease pathogeneses and thus provide useful clues for designing clinical studies but cannot adequately predict clinical outcomes, particularly in complex diseases such as COPD. Furthermore, anti-inflammatory and regenerative effects of MSCs likely depend on a number of intertwined factors including the disease state, local tissue environment, and MSC types. Thus, the urgently needed cell-based treatment for COPD necessitates further optimization of clinical trial protocols and employment of optimal MSC populations.

## 6. WJ-MSCs: A Promising Youthful Contender in Stem Cell Therapy for COPD

Mesenchymal stem cells derived from Wharton’s jelly (WJ-MSCs) are a primitive stromal cell population isolated from the umbilical cord [82]. WJ-MSCs are considered a favorable source of MSCs for the treatment of a range of diseases (including COPD) because of their unique properties useful for therapeutic applications [24,45,83,84]. These include their more primitive characteristics, abundant availability, lack of ethical concerns, noninvasive and painless collection, technically simple isolation, lack of teratogenicity and immunogenicity, and parity with BM-MSCs and AT-MSCs in terms of surface markers and cellular characteristics, albeit a higher proliferation capacity and longer life span. Novel findings reveal tissue specific and age/developmental stage-dependent phenotypical and functional differences among MSCs that seem critically important with respect to their utility in cell-based therapies, thus generating a widespread interest in **youthful** mesenchymal stem cells such as WJ-MSCs (for a recent review, see Kalaszczynska & Ferdyn, 2015 [84]). Specifically, the therapeutic potential of autologous BM-MSCs or AT-MSCs in the treatment of older patients (such as COPD patients) is apparently impaired by a number of age-related factors such as oxidative stress [85], telomere length [86], DNA damage [87], disease [88], and long-term use of some medications [89]. Accordingly, the youthful genotype and phenotype of neonatal tissue-derived MSCs, such as WJ-MSCs, has been associated with better adaptiveness to resident tissue environment and superior anti-inflammatory and immunomodulatory efficacy compared to mature MSCs [90,91,92].

Umbilical cords used to generate WJ-MSCs are typically obtained from healthy donors who have delivered babies following a full-term uncomplicated pregnancy [93]. While several techniques of WJ-MSC isolation from the umbilical cord have been reported, none has yet emerged as a standard [94,95]. Following isolation and expansion, WJ-MSCs are stored in liquid nitrogen with a shelf life of approximately 6 months. As an allogeneic but immune-privileged and “ready-made” cell product, WJ-MSCs can be applied straightaway/on demand within a time frame optimal for treatment.

Anti-inflammatory and tissue repair properties of mesenchymal cells derived from umbilical cord (UC-MSCs), including WJ-MSCs, have not been yet examined as extensively as those of BM-MSCs or AT-MSCs. However, available data from in vitro studies show that CB-MSCs express Clara cell secretory protein (CCSP), cystic fibrosis transmembrane conductance regulator (CFTR), surfactant protein C, thyroid transcription factor-1 mRNA, and both CCSP and CFTR protein [41]. Furthermore, WJ-MSCs limit neutrophil recruitment to inflamed endothelium through IL-6 [51], express epidermal growth factor (EGF), transforming growth factor-alpha (TGF-α), and their common cell-surface EGF/TGF-α -receptor [96], high levels of insulin-like growth factor (IGF)-1 and IGF-1 binding proteins [97], and molecules able to modulate NK cells and to expand Treg population [98].

Recent studies in animal models of lung disease have provided further mechanistic insights into the in vivo and in vitro effects of UC-MSCs/WJ-MSCs pertinent to potential MSC-based therapies for COPD. Specifically, in a bleomycin-induced mouse model of lung injury, WJ-MSCs reduced inflammation and inhibited the expression of TNF-α, interferon-γ, transforming growth factor-β, macrophage migratory inhibitory factor, and significantly reduced collagen concentration in the lung, presumably due to the simultaneous reduction in Smad2 phosphorylation (transforming growth factor-β activity), increased matrix metalloproteinase-2 levels, and reduced levels of their endogenous inhibitors [42]. Moreover, in Escherichia coli-induced acute lung injury in mice, transplantation of human WJ-MSCs attenuated lung injury and improved survival through the suppression of myeloperoxidase activity and proinflammatory cytokines (TNFα, IL-1α, IL-1β, IL-6, and macrophage inflammatory protein), which has been associated with the down-modulation of the chemotactic effects of neutrophils, immature dendrocytes, and NK cells, and the decreased chemotactic effects of T-cells [99]. Furthermore, in a rat model of lipopolysaccharide-induced lung injury, human UC-MSCs increased the survival rate and suppressed an increase of serum concentrations of TNF-α, IL-1β, and IL-6 without decreasing the level of anti-inflammatory cytokine IL-10 [100].

Since 2008, over 50 clinical trials registered at ClinicalTrials.gov [79] have employed WJ-MSCs in a wide range of therapeutic applications [84]. The already published results of some of these studies have demonstrated safety and efficacy of WJ-MSC systemic infusions in the treatment of various socially significant diseases such as type 1 and 2 diabetes mellitus [101,102], systemic lupus erythematosus [103], and late-onset hemorrhagic cystitis [104]. Currently, ClinicalTrials.gov [79] lists 17 clinical trials using MSCs in COPD patients (Table 2). However, safety and potential therapeutic effects of systemic administrations of WJ-MSCs in patients with COPD have yet to be examined.

## 7. Finding Optimal MSC Treatment Methodologies for COPD

Considering the post-application fate and therapeutic efficacy of exogenous MSCs, their delivery routes are of particular importance for medical applications (for a recent review, see Kean et al., 2013 [105]). Direct comparisons of the therapeutic effects of systemic (IV) vs. local (intratracheal; IT) MSC delivery in rodent models of lung disease are equivocal, i.e., show a similar efficacy of both methods [106] or total ineffectiveness of IT vs. IV injections [69]. However, beneficial effects of MSC transplantation using either IV or IT route have been reported in a number of preclinical studies (Table 1). In ClinicalTrials.com-listed clinical studies using MCSs in COPD, the vast majority (13 out of 17 with 1 unspecified) report the use of IV transplantations vs. two employing intrabronchial applications, and one using both IV and inhalation routes (Table 2). The major arguments for “local delivery” of MSCs are (1) safety; (2) avoidance of the pulmonary “first pass” effect [38,39]; and (3) an allocation of MSCs in the proximity of the injury site(s). However, no serious side effects has been reported in clinical trials using IV administration of MSCs in COPD patients [80,81] or clinical trials using IV administration of WJ-MSCs in the treatment of other diseases [101,102,103,104]. Furthermore, the pulmonary “first pass” entrapment effect as well as dispersed distribution of MSCs in the lungs are advantageous rather than inopportune in IV applications of MSCs in COPD patients. Taken together, the available data suggest that the systemic delivery of MSCs is the method of choice in the treatment of COPD, perhaps in combination with intrabronchial administration of MSCs as an adjuvant therapy.

Besides the selection of the most appropriate type of MSCs and their delivery route(s), the selection of the treatment group of patients who may benefit most from MSC treatment is clearly one of the key issues in cell-based therapies of various diseases, including COPD. The self-perpetuating and escalating character of the immune inflammatory processes underlying functional and structural changes in COPD, and the better-documented anti-inflammatory and protective vs. restorative actions of MSCs in animal models of COPD (Table 1), suggest that breaking the aforementioned vicious cycle at earlier stages of the disease should be more effective with respect to its progression. Likewise, younger patients may be more responsive to MSC treatment since elderly individuals do not respond to immune challenge as robustly as the young [107]. It is also worth considering in this context the promising results of studies using MSCs in animal models of COPD (Table 1) (which by design examine a selected aspect/mechanism of the disease usually unbiased by its multifaceted progression) vs. limited effects of MSC treatment of COPD in clinical trials completed so far [80,81]. It seems therefore that younger patients in the earlier stages of COPD are more likely to benefit from MSC therapy.

Based on the findings that several beneficial effects of MSCs are mediated by factors secreted by these cells, an emerging approach in MSC-based therapies is the use of media that are “conditioned” by MSCs via released microvesicles and secretomes such as cytokines, prostaglandins, or growth factors (for recent reviews, see [105,108]). Obviously, compared to MSCs, conditioned media (CM) are easier to produce, store, and apply. Accordingly, a rapidly increasing number of studies report their various therapeutic applications, although CM have not yet been tested in clinical trials. On the other hand, several studies indicate that cell-to-cell contact is essential for at least some actions of MSCs, including certain anti-inflammatory and immunomodulatory effects (Figure 1). Furthermore, unlike CM, MSCs appear to have the ability to home to target areas and, by sensing the environment, adapt their immunomodulatory or regenerative actions. Clearly, further studies are warranted regarding potential utility of CM in the treatment of COPD.

## 8. Alternative Cell-Based Therapies for COPD

Currently employed cell-based therapies for lung disease including COPD (for recent reviews, see Akram et al., 2016 [109] and Garcia et al., 2012 [110]) focus on three major areas: (1) transplantation of exogenous MSCs qualified to at least ameliorate the disease; (2) identifying and stimulating lung resident cell populations capable of responding to injury or disease; and (3) bioengineered 3D tissue constructs forming a transplantable lung organ. Harnessing the regenerative potential of resident lung stem/progenitor cells remains a promising yet poorly grasped process [111]. Considering the current status of lung tissue engineering [112,113], an attractive yet nascent potential approach in cases involving irreparable lung damage, its utility in the treatment of COPD in the foreseeable future seems unlikely. Thus, the major momentum of the present experimental and clinical studies of cell-based therapies points to improvement of lung function through transplantation of exogenous MSCs, perhaps in combination with an engagement of resident lung cells including resident stem/progenitor cells. 

It is well established that tissue-resident MSCs, including lung mesenchymal cells (LMCs), play a crucial role in tissue repair and regeneration. However, alterations in the number and/or phenotype of these cells have been implicated in fibrosis, inflammation, angiogenesis, and tumor formation, i.e., processes involved in the pathogenesis of a variety of lung diseases (for recent reviews, see Akram et al., 2016 [109], and Foronjy & Majka, 2012 [111]). Progress in the methods for isolation of progenitors from lung tissue, including discrete mesenchymal progenitor cell populations [114], has revealed a number of differentiated mesenchymal cell types (cartilage, smooth muscle, and myofibroblast) within the adult lung. It is worth noting in the context of complexity of LMCs’ actions that tumor-propagating cells require the inductive interaction of resident LMCs to foster lung cancer development [115]. Of particular interest with respect to dysfunctional lung remodeling in diseases such as COPD is maladaptive proliferation of vascular and myofibroblast cells consequent to altered pulmonary microenvironment [111]. Clearly, further studies are needed to fully understand the mechanisms of LMCs’ physiological function and impairment during disease, and thus to devise therapeutic strategies utilizing their regenerative potential. 

## 9. Conclusions

Transplantation of WJ-MSCs as a clinical alternative to other MSC-based therapies for various diseases, particularly immune inflammatory diseases such as COPD, is gaining widespread interest for a number of reasons including accessibility, higher expansion potential, low immunogenicity, immunoprotection, and greater agility of youthful vs. mature MSCs in anti-inflammatory and immunomodulatory actions. These features, combined with the safety of systemic infusions of WJ-MSCs demonstrated in several clinical trials in non-COPD disorders, provide a firm rationale for examining safety and efficiency of WJ-MSCs transplantation in patients with COPD, as well as for advancing translational research in this area.

## Figures and Tables

**Table 1:** In vivo and in vitro effects of mesenchymal stem cells (MSCs) in animal models of chronic obstructive pulmonary disease (COPD).

Animal/Model	MSC Type/Mode of Administration	Outcome/Potential Mechanism of Action	Reference
Rabbit elastase-induced	BM-MC/IT	↓ cell count in bronchoalveolar lavage fluid ↓ apoptotic cells and MMP-2 ↑ number of proliferative (Ki-67-positive) alveolar cells	Yuhgetsu et al., 2006 [57]
Mouse naphthalene-induced	BM-MSCs/IV	↑ regeneration of airway epithelial cells ↑ GFP transgene expression targeted delivery	Wong et al., 2007 [58]
Rat papain-induced	BM-MSCs/IT	↑ Bcl-2 and Bax differentiation of MSCs into type II alveolar epithelial cells ↓ alveolar cell apoptosis	Zhen et al., 2008 [59]
Mouse bleomycin-induced	hMSCs/IN	↓ TGFβ-1, MMIF, TNF-α ↓ collagen concentration in the lung ↓ Smad2 phosphorylation (transforming growth factor-beta activity) ↑ MMP2 ↓ fibrosis	Moodley et al., 2009 [42]
Rat papain-induced	BM-MSC/IV	↓ alveolar enlargement ↓ apoptosis ↑ VEGF-A	Zhen et al. 2010 [48]
Mouse elastase-induced	BM-MSC/IT	↑ HGF ↑ EGF ↑ SLPI	Katsha et al., 2010 [60]
Rat cigarette smoke-induced	BM-MC/IV	↓ apoptosis ↑ number of small pulmonary vessels ↓ pulmonary arterial pressure	Huh et al., 2011 [49]
Mouse cigarette smoke-induced	hAD-SC/IV mAD-SC/IV	↑ number of macrophages and polymorphonuclear leukocytes in the BAL, caspase-3 ↓ alveolar space size ↑ MAPK signal transduction pathways involved in inflammation and apoptosis ↓ VEGF	Schweitzer et al., 2011 [61]
Rat elastase-induced	AD-SC/SCI	↓ alveolar airspaces ↑ HGF, CINC-1 ↑ angiogenesis ↑ IL-1β	Furuya et al., 2012 [62]
Mouse elastase-induced	BM-MC/IV	↓ neutrophil infiltration, elastolysis, collagen fiber deposition ↓ lung cell apoptosis ↑ HGF, IGF ↓ PDGF, TGFβ-1, caspase-3	Cruz et al., 2012 [63]
Rat cigarette smoke-induced	BM-MSCs/IT hBM-MSCs in vitro	↓ TNF-α, IL-1β, MCP-1, IL-6 ↓ MMP9, MMP12 ↑ VEGF, VEGF receptor 2, TGFβ-1 ↓ pulmonary cell apoptosis	Guan et al., 2013 [64]
Rat LPS-induced	BM-MSCs/IV	↑ alveoli epithelial cells number	Zhao et al., 2014 [65]
Mouse elastase-induced	BM-MSCs, AD-MSC or lung tissue (LMSC)/IV/IT	↓ neutrophil infiltration, cell apoptosis ↑ elastic fiber content ↓ alveolar epithelial and endothelial cell damage ↓ keratinocyte-derived chemokine (KC, a mouse analog of interleukin-8), TGFβ-1 ↓ alveolar hyperinflation (BM-MSCs), collagen fiber content (BM-MSCs and L-MSC) ↓ M1 macrophages and pulmonary hypertension ↑ VEGF	Antunes at al., 2014 [66]
Rat cigarette smoke-induced	iPSC-MSCs/IV BM-MSCs/IV	↑ adenosine triphosphate	Li et al., 2014 [67]
Guinea pig cigarette smoke-induced	ADSCs/IV/IT	↑ antioxidant effects ↓ apoptotic cells ↓ oxidative damage weight restoration	Ghorbani et al., 2014 [68]
Mouse elastase-induced	BM-MSCs/IV/IT	↑ IL-6, keratinocyte-derived-chemokine (KC) ↓ MCP levels at day 2 after elastase injection	Tibboel et al., 2014 [69]
Mouse DDMC, non-viral vector	hMSCs/IT	↓ emphysema ↑ cells within the lung parenchyma	Zarogoulidis et al., 2014 [70]
Rat cigarette smoke-induced	AFMSCs/IT	↑ SPA, ↑ SPC ↑ TTF-1 ↓ AECII apoptosis ↓ lung injury	Li et al., 2014 [71]
Rat cigarette smoke-induced	BM-MSCs/IT	↓ COX-2, COX-2-mediated prostaglandin E2 (PGE2) in macrophages through inhibition of phosphorylation of p38 MAPK and ERK-activation	Gu et al., 2015 [72]
Mouse elastase-induced	BD-MSCs/IV	↓ VEGF-A ↓ HO-1	Chen et al., 2015 [73]
Mouse elastase-induced	hMSCs/IV	↓ MMP-9 ↑ VEGF ↑ IL-1β, INF-γ, IL-2	Kim et al., 2015 [74]
Mouse cigarette smoke-induced	htMSCs/IV	↓ lung inflammation ↓ IL-1β, IL-6, TNF-α, KC - (C-X-C motif) ligand 1 (CXCL1) ↓ mucus production, collagen accumulation, tissue damage ↓ NF-κB ↑ IL-10	Peron et al., 2015 [75]
Rat elastase-induced	ADSCs/IV	↑ HGF expression in lung tissues ↑ alveolar and vascular regeneration ↓ alveolar cell apoptosis ↑ VEGF, HGF, bFGF	Shigemura et al., 2016 [76]
Mouse elastase-induced	hBM-MSCs/IV	↑ HGF	Kennelly et al., 2016 [77]

ET-1: Endothelin-1; HO-1: heme oxygenase-1; IN: intranasal; IT: intratracheally; IGF: insulin-like growth factor; iNOS: inducible NOS; IV: intravenous; OA: oropharyngeal aspiration; STAT: signal transducer and activator of transcription; TSG-6: transcription; TSG-6: tumor necrosis factor alpha-induced protein 6; LPS: lipopolysaccharide; Bcl-2: B-cell lymphoma 2; BM-MC: bone marrow mononuclear derived cells; BM-MSC: bone marrow-derived mesenchymal stem cells; ADSC: adipose-derived stem cell; hBM-MSCs: human bone marrow-derived mesenchymal stem cells; hMSCs: human umbilical cord cells derived from Wharton’s jelly; iPSC-MSCs: human-induced pluripotent stem cell-derived MSCs; AFMSCs: amniotic fluid-derived mesenchymal stromal cells; htMSCs: human tubal-derived mesenchymal stromal cells; hAD-SC: human adult adipose-derived stromal (stem) cells; mAD-SC: mouse adult adipose-derived stromal (stem) cells; IL-1β: interleukin; INF-γ: interferon γ; VEGF-A: vascular endothelial growth factor A; HGF: endogenous hepatocyte growth factor; VEGF: vascular endothelial growth factor; bFGF: basic fibroblast growth factor; MMP-2: matrix metalloproteinase-2; MMP9 matrix metalloproteinase-9; MMP12: matrix metalloproteinase-12; TGFβ-1: transforming growth factor β; CINC-1: cytokine-induced neutrophil chemoattractant; EGF: epidermal growth factor; SLPI: secretory leukocyte protease inhibitor; TTF-1: thyroid transcription factor 1; TNF-α: tumor necrosis factor-alpha; COX-2: cyclooxygenase-2; TGFβ-1-transformig growth factor-beta; IFNG: interferon-gamma; MMIF: macrophage migratory inhibitory factor; PDGF: platelet-derived growth factor; IGF: insulin growth factor.

**Table 2:** Clinical trials using MSC therapies in COPD listed at ClinicalTrials.gov [79] as of 17 October 2017.

Nct Number	Time Frame	Msctype	Msc Source	Delivery Route	MSC Dose	Application Schedule	No. of Patients	Follow-up Period	Trial Status	Studyphase	Study Location
NCT00683722	2008–2010	BM-MSC	autologous	IV	1 × 10^8^	Four monthly	62	2 years	Completed	II	USA
NCT01110252	2010–2011	BM-MC	autologous	IV	1 × 10^8^	Single dose	4	1 year	Completed	*	Brazil
NCT01306513	2010–2012	BM-MSC	autologous	IV	*	Twice weekly	10	8 weeks	Completed	I	Netherlands
NCT01849159	2014–2017	BM-MSC	autologous	IV	2 × 10^8^	Every 2 mo for 1 year	30	2 years	Unknown	I/II	Russia
NCT01872624	2013–2015	BM-MSC	autologous	EB	*	Single dose	10	4 months	Completed	*	Brazil
NCT01758055	2012–2014	BM-MC	autologous	EB	60 × 10^6^	Single dose	12	1 year	Unknown	I	Iran
NCT02041000	2014	ADSC	autologous	*	*	*	0	6 months	Withdrawn	*	USA
NCT01559051	2014–2017	AD-SVF	autologous	IV/IN	*	Single dose	100	6 months	Recruiting	I/II	USA
NCT02161744	2014–2017	AD-SVF	autologous	IV	*	Single dose	60	1 year	Recruiting	I	USA
NCT02216630	2014–2017	AD-SVF	autologous	IV	*	Single dose	26	1 year	Completed	I/II	USA
NCT02135380	2014–2015	AD-SVF	autologous	IV	*	Single dose	60	1 year	Unknown	I/II	India
		AD-MSC	autologous	IV	2 × 10^6^/kg b.m.	Three doses					
NCT02645305	2015–2016	ADSC & PRP	autologous	IV	*	Single dose	20	1 year	Recruiting	I/II	Vietnam
NCT02348060	2015–2018	AD-SVF	autologous	IV	*	Single dose	75	1 year	Recruiting	*	USA
NCT03044431	2016–2018	BM-MC & PRP	autologous	IV	*	Single dose	214	6 months	Active, not recruiting	*	USA
NCT02412332	2015–2017	BM-MC	autologous	IV	1 × 10^8^	Single dose	20	1 year	Enrolling	I/II	Brazil
		ADSC			1 × 10^8^						
		BM-MC +ADSC			5 × 10^7^ + 5 × 10^7^						
NCT03228121	2017–2018	PBSC & PRP	autologous	IV	*	Three doses	100	1 year	Enrolling	*	USA

BM-MC: bone marrow mononuclear-derived; BM-MSC: bone marrow-derived mesenchymal stem cells, ADSC: adipose-derived stem cell; AD-SVF: adipose-derived stromal vascular faction; AD-MSC: adipose-derived mesenchymal stem/stroma; PRP: palate-rich plasma; PBSC: peripheral blood stem cells; IV: intravenous; EB: endobronchial; IN: inhalation; * data not provided.

## References

[1] Strategy for the Diagnosis, Management and Prevention of COPD (2017). Global Initiative for Chronic Obstructive Lung Disease (GOLD). http://goldcopd.org.

[2] Lozano R., Naghavi M., Foreman K., Lim S., Shibuya K., Aboyans V., Abraham J., Adair T., Aggarwal R., Ahn S.Y. (2012). Global and regional mortality from 235 causes of death for 20 age groups in 1990 and 2010: A systematic analysis for the Global Burden of Disease Study 2010. Lancet.

[3] Chung K.F., Adcock I.M. (2008). Multifaceted mechanisms in COPD: Inflammation, immunity, and tissue repair and destruction. Eur. Respir. J..

[4] Tuder R.M., Petrache I. (2012). Pathogenesis of chronic obstructive pulmonary disease. J. Clin. Investig..

[5] Bagdonas E., Raudoniute J., Bruzauskaite I., Aldonyte R. (2015). Novel aspects of pathogenesis and regeneration mechanisms in COPD. Int. J. Chronic Obstr. Pulm. Dis..

[6] Chen Z.-H., Kim H.P., Ryter S.W., Cho A.M.K. (2008). Identifying targets for COPD treatment through gene expression analyses. Int. J. Chronic Obstr. Pulm. Dis..

[7] Molfino N.A., Coyle A.J. (2008). Gene-environment interactions in chronic obstructive pulmonary disease. Int. J. Chronic Obstr. Pulm. Dis..

[8] Fischer B.M., Pavlisko E., Voynow J.A. (2011). Pathogenic triad in COPD: Oxidative stress, protease-antiprotease imbalance, and inflammation. Int. J. Chronic Obstr. Pulm. Dis..

[9] Shapiro S.D. (2001). End-stage chronic obstructive pulmonary disease. Am. J. Respir. Crit. Care Med..

[10] Ind P.W. (2005). COPD disease progression and airway inflammation: Uncoupled by smoking cessation. Eur. Respir. J..

[11] Donaldson G.C., Seemungal T.A.R., Patel I.S., Bhowmik A., Wilkinson T.M., Hurst J.R., Maccallum P.K., Wedzicha J.A. (2005). Airway and systemic inflammation and decline in lung function in patients with COPD. Chest.

[12] Rovina N., Koutsoukou A., Koulouris N.G. (2013). Inflammation and Immune Response in COPD: Where do we stand?. Mediat. Inflamm..

[13] Barnes P.J. (2003). New concepts in chronic obstructive pulmonary disease. Annu. Rev. Med..

[14] Boer W., Alagappan V., Sharma H. (2007). Molecular mechanisms in chronic obstructive pulmonary disease. Cell Biochem. Biophys..

[15] Hogg J.C., Timens W. (2009). The pathology of chronic obstructive pulmonary disease. Annu. Rev. Pathol..

[16] Wecht S., Rojas M. (2016). Mesenchymal stem cells in the treatment of chronic lung disease. Respirology.

[17] Chung E., Son Y. (2014). Crosstalk between mesenchymal stem cells and macrophages in tissue repair. Tissue Eng. Regen. Med..

[18] Williams T.J., Jose P.J. (2001). Neutrophils in chronic obstructive pulmonary disease. Novartis Found. Symp..

[19] Barnes P.J., Cosio M.G. (2004). Characterization of T lymphocytes in chronic obstructive pulmonary disease. PLoS Med..

[20] Sharafkhaneh A., Hanania N.A., Kim V. (2008). Pathogenesis of emphysema: From the bench to the bedside. Proc. Am. Thorac. Soc..

[21] Hogg J.C., Paré P.D., Hackett T.L. (2017). The contribution of small airway obstruction to the pathogenesis of chronic obstructive pulmonary disease. Physiol. Rev..

[22] Chamberlain G., Fox J., Ashton B., Middleton J. (2007). Concise review: Mesenchymal stem cells: Their phenotype, differentiation capacity, immunological features, and potential for homing. Stem Cells.

[23] Dominici M., le Blanc K., Mueller I., Slaper-Cortenbach I., Marini F., Krause D., Deans R., Keating A., Prockop D.J., Horwitz E. (2006). Minimal criteria for defining multipotent mesenchymal stromal cells. The International Society for Cellular Therapy position statement. Cytotherapy.

[24] Ostanin A.A., Petrovskii Y.L., Sheleva E.Y., Chernykh E.R. (2011). Multiplex analysis of cytokines, chemokines, growth factors, MMP-9 and TIMP-1 produced by human bone marrow, adipose tissue, and placental mesenchymal stromal cells. Bull. Exp. Biol. Med..

[25] Ryan J.M., Barry F.P., Murphy J.M., Mahon B.P. (2005). Mesenchymal stem cells avoid allogeneic rejection. J. Inflamm..

[26] English K. (2013). Mechanisms of mesenchymal stromal cell immunomodulation. Immunol. Cell Biol..

[27] Spees J.L., Lee R.H., Gregory C.A. (2016). Mechanisms of mesenchymal stem/stromal cell function. Stem Cell Res. Ther..

[28] Moodley Y., Manuelpillai U., Weiss D. (2011). Cellular therapies for lung disease: A distant Horizon. Respirology.

[29] Inamdar A.C., Inamdar A.A. (2013). Mesenchymal stem cell therapy in lung disorders: Pathogenesis of lung diseases and mechanism of action of mesenchymal stem cell. Exp. Lung Res..

[30] Conese M., Carbone A., Castellani S., di Gioia S. (2013). Paracrine effects and heterogeneity of marrow-derived stem/progenitor cells: Relevance for the treatment of respiratory diseases. Cells Tissues Organs.

[31] Weiss D.J. (2014). Current status of stem cells and regenerative medicine in lung biology and diseases. Stem Cells.

[32] Gao F., Chiu M., Motan D.A.L., Zhang Z., Chen L., Ji H.-L., Tse H.-F., Fu Q.-L., Lian Q. (2016). Mesenchymal stem cells and immunomodulation: Current status and future prospects. Cell Death Dis..

[33] Le Blanc K., Ringden O. (2007). Immunomodulation by mesenchymal stem cells and clinical experience. J. Intern. Med..

[34] Wang L.-T., Ting C.-H., Yen M.-L., Liu K.J., Sytwu H.K., Wu K.K., Yen B.L. (2016). Human mesenchymal stem cells (MSCs) for treatment towards immune- and inflammation-mediated diseases: Review of current clinical trials. J. Biomed. Sci..

[35] Cheng S.-L., Lin C.-H., Yao C.-L. (2017). Mesenchymal stem cell administration in patients with chronic obstructive pulmonary disease: State of the science. Stem Cells Int..

[36] Kang S.K., Shin I.S., Ko M.S., Jo J.Y., Ra J.C. (2012). Journey of mesenchymal stem cells for homing: Strategies to enhance efficacy and safety of stem cell therapy. Stem Cells Int..

[37] Karp J.M., Leng Teo G.S. (2009). Mesenchymal stem cell homing: The devil is in the details. Cell Stem Cell.

[38] Schrepfer S., Deuse T., Reichenspurner H., Fischbein M.P., Robbins R.C., Pelletier M.P. (2007). Stem cell transplantation: The lung barrier. Transplant. Proc..

[39] Barbash I.M., Chouraqui P., Baron J., Feinberg M.S., Etzion S., Tessone A., Miller L., Guetta E., Zipori D., Kedes L.H. (2003). Systemic delivery of bone marrow-derived mesenchymal stem cells to the infarcted myocardium: Feasibility, cell migration, and body distribution. Circulation.

[40] Loi R., Beckett T., Goncz K.K., Suratt B.T., Weiss D.J. (2006). Limited restoration of cystic fibrosis lung epithelium in vivo with adult bone marrow-derived cells. Am. J. Respir. Crit. Care Med..

[41] Sueblinvong V., Loi R., Eisenhauer P.L., Bernstein I.M., Suratt B.T., Spees J.L., Weiss D.J. (2008). Derivation of lung epithelium from human cord blood-derived mesenchymal stem cells. Am. J. Respir. Crit. Care Med..

[42] Moodley Y., Atienza D., Manuelpillai U., Samuel C.S., Tchongue J., Ilancheran S., Boyd R., Trounson A. (2009). Human umbilical cord mesenchymal stem cells reduce fibrosis of bleomycin-induced lung injury. Am. J. Pathol..

[43] Liu A.R., Liu L., Chen S., Yang Y., Zhao H.J., Liu L., Guo F.M., Lu X.M., Qiu H.B. (2013). Activation of canonical wnt pathway promotes differentiation of mouse bone marrow-derived MSCs into type II alveolar epithelial cells, confers resistance to oxidative stress, and promotes their migration to injured lung tissue in vitro. J. Cell. Physiol..

[44] Yagi H., Soto-Gutierrez A., Parekkadan B., Kitagawa Y., Tompkins R.G., Kobayashi N., Yarmush M.L. (2010). Mesenchymal stem cells: Mechanisms of immunomodulation and homing. Cell Transplant..

[45] Hass R., Kasper C., Bohm S., Jacobs R. (2011). Different populations and sources of human mesenchymal stem cells (MSC): A comparison of adult and neonatal tissue-derived MSC. Cell Commun. Signal..

[46] Nemeth K., Leelahavanichkul A., Yuen P.S., Mayer B., Parmelee A., Doi K., Robey P.G., Leelahavanichkul K., Koller B.H., Brown J.M. (2009). Bone marrow stromal cells attenuate sepsis via prostaglandin E2-dependent reprogramming of host macrophages to increase their interleukin-10 production. Nat. Med..

[47] Gupta N., Su X., Popov B., Lee J.W., Serikov V., Matthay M.A. (2007). Intrapulmonary delivery of bone marrow-derived mesenchymal stem cells improves survival and attenuates endotoxin-induced acute lung injury in mice. J. Immunol..

[48] Zhen G., Xue Z., Zhao J., Gu N., Tang Z., Xu Y., Zhang Z. (2010). Mesenchymal stem cell transplantation increases expression of vascular endothelial growth factor in papain-induced emphysematous lungs and inhibits apoptosis of lung cells. Cytotherapy.

[49] Huh J.W., Kim S.Y., Lee J.H., Lee J.S., Van Ta Q., Kim M., Oh Y.M., Lee Y.S., Lee S.D. (2011). Bone marrow cells repair cigarette smoke-induced emphysema in rats. Am. J. Physiol. Lung Cell. Mol. Physiol..

[50] Raffaghello L., Bianchi G., Bertolotto M., Montecucco F., Busca A., Dallegri F., Ottonello L., Pistoia V. (2008). Human mesenchymal stem cells inhibit neutrophil apoptosis: A model for neutrophil preservation in the bone marrow niche. Stem Cells.

[51] Munir H., Luu N.T., Clarke L.S., Nash G.B., McGettrick H.M. (2016). Comparative ability of mesenchymal stromal cells from different tissues to limit neutrophil recruitment to inflamed endothelium. PLoS ONE.

[52] Han K.H., Ro H., Hong J.H., Lee E.M., Cho B., Yeom H.J., Kim M.G., Oh K.H., Ahn C., Yang J. (2011). Immunosuppressive mechanisms of embryonic stem cells and mesenchymal stem cells in alloimmune response. Transplant. Immunol..

[53] Krampera M., Glennie S., Dyson J., Scott D., Laylor R., Simpson E., Dazzi F. (2003). Bone marrow mesenchymal stem cells inhibit the response of naive and memory antigen-specific T cells to their cognate peptide. Blood.

[54] Sotiropoulou P.A., Perez S.A., Gritzapis A.D., Baxevanis C.N., Papamichail M. (2006). Interactions between human mesenchymal stem cells and natural killer cells. Stem Cells.

[55] Sheng H., Wang Y., Jin Y., Zhang Q., Zhang Y., Wang L., Shen B., Yin S., Liu W., Cui L. (2008). A critical role of IFN γ in priming MSC-mediated suppression of T cell proliferation through up-regulation of B7-H1. Cell Res..

[56] Ren G., Zhao X., Zhang L., Zhang J., L’Huillier A., Ling W., Roberts A.I., Le A.D., Shi S., Shao C. (2010). Inflammatory cytokine-induced intercellular adhesion molecule-1 and vascular cell adhesion molecule-1 in mesenchymal stem cells are critical for immunosuppression. J. Immunol..

[57] Yuhgetsu H., Ohno Y., Funaguchi N., Asai T., Sawada M., Takemura G., Minatoguchi S., Fujiwara H., Fujiwara T. (2006). Beneficial effects of autologous bone marrow mononuclear cell transplantation against elastase-induced emphysema in rabbit. Exp. Lung Res..

[58] Wong A.P., Dutly A.E., Sacher A., Lee H., Hwang D.M., Liu M., Keshavjee S., Hu J., Waddell T.K. (2007). Targeted cell replacement with bone marrow cells for airway epithelial regeneration. Am. J. Physiol. Lung Cell. Mol. Physiol..

[59] Zhen G., Liu H., Gu N., Zhang H., Xu Y., Zhang Z. (2008). Mesenchymal stem cells transplantation protects against rat pulmonary emphysema. Front. Biosci..

[60] Katsha A.M., Ohkouchi S., Xin H., Kanehira M., Sun R., Nukiwa T., Saijo Y. (2010). Paracrine factors of multipotent stromal cells ameliorate lung injury in an elastase-induced emphysema model. Mol. Ther..

[61] Schweitzer K.S., Johnstone B.H., Garrison J., Rush N.I., Cooper S., Traktuev D.O., Feng D., Adamowicz J.J., van Demark M., Fisher A.J. (2011). Adipose stem cell treatment in mice attenuates lung and systemic injury induced by cigarette smoking. Am. J. Respir. Crit. Care Med..

[62] Furuya N., Takenaga M., Ohta Y., Tokura Y., Hamaguchi A., Sakamaki A., Kida H., Handa H., Nishine H., Mineshita M. (2012). Cell therapy with adipose tissue-derived stem/stromal cells for elastase-induced pulmonary emphysema in rats. Regen. Med..

[63] Cruz F.F., Antunes M.A., Abreu S.C., Fujisaki L.C., Silva J.D., Xisto D.G., Maron-Gutierrez T., Ornellas D.S., Sá V.K., Rocha N.N. (2012). Protective effects of bone marrow mononuclear cell therapy on lung and heart in an elastase-induced emphysema model. Respir. Physiol. Neurobiol..

[64] Guan X.J., Song L., Han F.F., Cui Z.L., Chen X., Guo X.J., Xu W.G. (2013). Mesenchymal stem cells protect cigarette smoke-damaged lung and pulmonary function partly via VEGF-VEGF receptors. J. Cell. Biochem..

[65] Zhao Y., Xu A., Xu Q., Zhao W., Li D., Fang X., Ren Y. (2014). Bone marrow mesenchymal stem cell transplantation for treatment of emphysemic rats. Int. J. Clin. Exp. Med..

[66] Antunes M.A., Abreu S.C., Cruz F.F., Teixeira A.C., Lopes-Pacheco M., Bandeira E., Olsen P.C., Diaz B.L., Takyia C.M., Freitas I.P. (2014). Effects of different mesenchymal stromal cell sources and delivery routes in experimental emphysema. Respir. Res..

[67] Li X., Zhang Y., Yeung S.C., Liang Y., Liang X., Ding Y., Ip M.S., Tse H.F., Mak J.C., Lian Q. (2014). Mitochondrial transfer of induced pluripotent stem cell-derived mesenchymal stem cells to airway epithelial cells attenuates cigarette smoke-induced damage. Am. J. Respir. Cell Mol. Biol..

[68] Ghorbani A., Feizpour A., Hashemzahi M., Gholami L., Hosseini M., Soukhtanloo M., Bagheri F.V., Khodaei E., Roshan N.M., Boskabady M.H. (2014). The effect of adipose derived stromal cells on oxidative stress level, lung emphysema and white blood cells of guinea pigs model of chronic obstructive pulmonary disease. Daru.

[69] Tibboel J., Keijzer R., Irwin Reiss I., de Jongste J.C., Post M. (2014). Intravenous and intratracheal mesenchymal stromal cell injection in a mouse model of pulmonary emphysema. Int. J. Chronic Obstr. Pulm. Dis..

[70] Zarogoulidis P., Hohenforst-Schmidt W., Huang H., Sahpatzidou D., Freitag L., Sakkas L., Rapti A., Kioumis I., Pitsiou G., Kouzi-Koliakos K. (2014). A gene therapy induced emphysema model and the protective role of stem cells. Diagn. Pathol..

[71] Li Y., Gu C., Xu W., Yan J., Xia Y., Ma Y., Chen C., He X., Tao H. (2014). Therapeutic effects of amniotic fluid-derived mesenchymal stromal cells on lung injury in rats with emphysema. Respir. Res..

[72] Gu W., Song L., Li X.M., Di W., Wang X.J., Xu W.G. (2015). Mesenchymal stem cells alleviate airway inflammation and emphysema in COPD through down-regulation of cyclooxygenase-2 (COX-2) via p38 and ERK MAPK pathways. Sci. Rep..

[73] Chen Y.B., Lan Y.W., Chen L.G., Huang T.T., Choo K.B., Cheng W.T., Lee H.S., Chong K.Y. (2015). Mesenchymal stem cell-based HSP70 promoter-driven VEGFA induction by resveratrol alleviates elastase-induced emphysema in a mouse model. Cell Stress Chaperones.

[74] Kim Y.S., Kim J.Y., Huh J.W., Lee S.W., Choi S.J., Oh Y.M. (2015). The therapeutic effects of optimal dose of mesenchymal stem cells in a murine model of an elastase induced-emphysema. Tuberc. Respir. Dis..

[75] Peron J.P., de Brito A.A., Pelatti M., Brandão W.N., Vitoretti L.B., Greiffo F.R., da Silveira E.C., Oliveira-Junior M.C., Maluf M., Evangelista L. (2015). Human tubal-derived mesenchymal stromal cells associated with low level laser therapy significantly reduces cigarette smoke-induced COPD in C57BL/6 mice. PLoS ONE.

[76] Shigemura N., Okumura M., Mizuno S., Imanishi Y., Nakamura T., Sawa Y. (2006). Autologous transplantation of adipose tissue-derived stromal cells ameliorates pulmonary emphysema. Am. J. Transplant..

[77] Kennelly H., Mahon B.P., English K. (2016). Human mesenchymal stromal cells exert HGF dependent cytoprotective effects in a human relevant pre-clinical model of COPD. Sci. Rep..

[78] Antunes M.A., Laffey J.G., Pelosi P., Rocco P.R.M. (2014). Mesenchymal stem cell trials for pulmonary diseases. J. Cell. Biochem..

[79] ClinicalTrials.Gov. https://clinicaltrials.gov.

[80] Weiss D.J., Casaburi R., Flannery R., LeRoux-Williams M., Tashkin D.P. (2013). A placebo-controlled, randomized trial of mesenchymal stem cells in COPD. Chest.

[81] Stessuk T., Ruiz M.A., Greco O.T., Bilaqui A., de Oliveira Ribeiro-Paes M.J., Ribeiro-Paes J.T. (2013). Phase I clinical trial of cell therapy in patients with advanced chronic obstructive pulmonary disease: Follow-up for up to 3 years. Rev. Bras. Hematol. Hemoter..

[82] Wang H.S., Hung S.C., Peng S.T., Huang C.C., Wei H.M., Guo Y.J., Fu Y.S., Lai M.C., Chen C.C. (2004). Mesenchymal stem cells in the Wharton’s jelly of the human umbilical cord. Stem Cells.

[83] Pappa K.I., Anagnou N.P. (2009). Novel sources of fetal stem cells: Where do they fit on the developmental continuum?. Regen. Med..

[84] Kalaszczynska I., Ferdyn K. (2015). Wharton’s jelly derived mesenchymal stem cells: Future of regenerative medicine? Recent findings and clinical significance. BioMed Res. Int..

[85] Stolzing A., Jones E., McGonagle D., Scutt A. (2008). Age-related changes in human bone marrow-derived mesenchymal stem cells: Consequences for cell therapies. Mech. Ageing Dev..

[86] Van Zglinicki T., Saretzki G., Docke W., Lotze C. (1995). Mild hyperoxia shortens telomeres and inhibits proliferation: A model for senescence?. Exp. Cell Res..

[87] Li T., Zhou Z.W., Ju Z., Wang Z.Q. (2016). DNA damage response in hematopoietic stem cell ageing. Genom. Proteom. Bioinform..

[88] Oñate B., Vilahur G., Camino-López S., Díez-Caballero A., Ballesta-López C., Ybarra J., Moscatiello F., Herrero J., Badimon L. (2013). Stem cells isolated from adipose tissue of obese patients show changes in their transcriptomic profile that indicate loss in stemcellness and increased commitment to an adipocyte-like phenotype. BMC Genom..

[89] Müller M., Raabe O., Addicks K., Wenisch S., Arnhold S. (2011). Effects of non-steroidal anti-inflammatory drugs on proliferation, differentiation and migration in equine mesenchymal stem cells. Cell Biol. Int..

[90] Baksh D., Yao R., Tuan R.S. (2007). Comparison of proliferative and multilineage differentiation potential of human mesenchymal stem cells derived from umbilical cord and bone marrow. Stem Cells.

[91] Wu L.F., Wang N.N., Liu Y.S., Wei X. (2009). Differentiation of Wharton’s jelly primitive stromal cells into insulin-producing cells in comparison with bone marrow mesenchymal stem cells. Tissue Eng. Part A.

[92] Prasanna S.J., Jahnavi V.S. (2011). Wharton’s jelly mesenchymal stem cells as off-the-shelf cellular therapeutics: A closer look into their regenerative and immunomodulatory properties. Open Tissue Eng. Regen. Med. J..

[93] Riezzo I., Pascale N., la Russa R., Liso A., Salerno M., Turillazzi E. (2017). Donor selection for allogenic hemopoietic stem cell transplantation: Clinical and ethical considerations. Stem Cells Int..

[94] Smith J.R., Pfeifer K., Petry F., Powell N., Delzeit J., Weiss M. (2016). Standardizing umbilical cord mesenchymal stromal cells for translation to clinical use: Selection of GMP Compliant medium and a simplified isolation method. Stem Cells Int..

[95] Smith J.R., Cromer A., Weiss M.L. (2017). Human umbilical cord mesenchymal stromal cell isolation, expansion, cryopreservation, and characterization. Curr. Protoc. Stem Cell Biol..

[96] Rao C.V., Li X., Toth P., Lei Z.M. (1995). Expression of epidermal growth factor, transforming growth factor-α, and their common receptor genes in human umbilical cords. J. Clin. Endocrinol. Metab..

[97] Palka J., Bankowski E., Jaworski S. (2000). An accumulation of IGF-I and IGF-binding proteins in human umbilical cord. Mol. Cell. Biochem..

[98] Anzalone R., lo Iacono M., Corrao S., Magno F., Loria T., Cappello F., Zummo G., Farina F., la Rocca G. (2010). New emerging potentials for human Wharton’s jelly mesenchymal stem cells: Immunological features and hepatocyte-like differentiative capacity. Stem Cells Dev..

[99] Kim E.S., Chang Y.S., Choi S.J., Kim J.K., Yoo H.S., Ahn S.Y., Sung D.K., Kim S.Y., Park Y.R., Park W.S. (2011). Intratracheal transplantation of human umbilical cord blood-derived mesenchymal stem cells attenuates Escherichia coli-induced acute lung injury in mice. Respir. Res..

[100] Li J., Li D., Liu X., Tang S., Wei F. (2012). Human umbilical cord mesenchymal stem cells reduce systemic inflammation and attenuate LPS-induced acute lung injury in rats. J. Inflamm..

[101] Hu J., Yu X., Wang Z., Wang F., Wang L., Gao H., Chen Y., Zhao W., Jia Z., Yan S. (2013). Long term effects of the implantation of Wharton’s jelly-derived mesenchymal stem cells from the umbilical cord for newly-onset type 1 diabetes mellitus. Endocr. J..

[102] Liu X., Zheng P., Wang X., Dai G., Cheng H., Zhang Z., Hua R., Niu X., Shi J., An Y. (2014). A preliminary evaluation of efficacy and safety of Wharton’s jelly mesenchymal stem cell transplantation in patients with type 2 diabetes mellitus. Stem Cell Res. Ther..

[103] Wang D., Li J., Zhang Y., Zhang M., Chen J., Li X., Hu X., Jiang S., Shi S., Sun L. (2014). Umbilical cord mesenchymal stem cell transplantation in active and refractory systemic lupus erythematosus: A multicenter clinical study. Arthritis Res. Ther..

[104] Wang Y., Chen F., Gu B., Chen G., Chang H., Wu D. (2015). Mesenchymal stromal cells as an adjuvant treatment for severe late-onset hemorrhagic cystitis after allogeneic hematopoietic stem cell transplantation. Acta Haematol..

[105] Kean T.J., Lin P., Caplan A.I., Dennis J.E. (2013). MSCs: Delivery routes and engraftment, cell-targeting strategies, and immune modulation. Stem Cells Int..

[106] Curley G.F., Ansari B., Hayes M., Devaney J., Masterson C., Ryan A., Barry F., O’Brien T., Toole D.O., Laffey J.G. (2013). Effects of intratracheal mesenchymal stromal cell therapy during recovery and resolution after ventilator-induced lung injury. Anesthesiology.

[107] Montecino-Rodriguez E., Berent-Maoz B., Dorshkind K. (2013). Causes, consequences, and reversal of immune system aging. J. Clin. Investig..

[108] Pawitan J.A. (2014). Prospect of stem cell conditioned medium in regenerative medicine. BioMed Res. Int..

[109] Akram K.M., Patel N., Spiteri M.A., Forsyth N.R. (2016). Lung Regeneration: Endogenous and Exogenous Stem Cell Mediated Therapeutic Approaches. Int. J. Mol. Sci..

[110] Garcia O., Carraro G., Navarro S., Bertoncello I., McQualter J., Driscoll B., Jesudason E., Warburton D. (2012). Cell-based therapies for lung disease. Br. Med. Bull..

[111] Foronjy R.F., Majka S.M. (2012). The potential for resident lung mesenchymal stem cells to promote functional tissue regeneration: Understanding microenvironmental cues. Cells.

[112] O’Leary C., Gilbert J.L., O’Dea S., O’Brien F.J., Cryan S.A. (2015). Respiratory Tissue Engineering: Current Status and Opportunities for the Future. Tissue Eng. Part B Rev..

[113] Schilders K.A.A., Eenjes E., van Riet S., Poot A.A., Stamatialis D., Truckenmüller R., Hiemstra P.S., Rottier R.J. (2016). Regeneration of the lung: Lung stem cells and the development of lung mimicking devices. Respir. Res..

[114] Summer R., Fitzsimmons K., Dwyer D., Murphy J., Fine A. (2007). Isolation of an adult mouse lung mesenchymal progenitor cell population. Am. J. Respir. Cell Mol. Biol..

[115] Ampollini L., Madeddu D., Falco A., Frati C., Lorusso B., Graiani G., Saccani F., Gervasi A., Rossetti P., Bonomini S. (2014). Lung mesenchymal cells function as an inductive microenvironment for human lung cancer propagating cells dagger. Eur. J. Cardiothorac. Surg..

